# Body and mind: how somatic feedback signals shape brain activity and cognition

**DOI:** 10.1007/s00424-022-02778-5

**Published:** 2022-12-12

**Authors:** Andreas Draguhn, Jonas F. Sauer

**Affiliations:** 1 Institut für Physiologie und Pathophysiologie, Medizinische Fakultät der Universität Heidelberg, Universität Heidelberg, Heidelberg, Germany; 2 Physiologisches Institut I, Albert-Ludwigs-Universität Freiburg, Freiburg, Germany

Nothing in Biology Makes Sense Except in the Light of Evolution [10]

A major aim of modern neuroscience is the causal explanation of behavioral and cognitive processes by their underlying neuronal mechanisms. This research program has become increasingly successful due to strong methodological advances, including functional imaging and massively parallel electrophysiological recordings, sophisticated behavioral models, interventional techniques like opto- and chemogenetics, large data collections and advanced methods of analysis. Many of these explanations conceptualize cognition, explicitly or implicitly, as ‘information processing’, i.e. the extraction of relevant features and patterns, comparison with predictions, creation of mental representations, etc. This research program has been criticized by different scientists during past decades because it does not sufficiently account for the embedding of the brain in the organism, and the organism in its environment [9, 34]. Theorists of ‘embodied cognition’ claim that we need to focus on the mutual interactions between all parts of living organisms and their environment.

The shortcomings of dis-embodied models of cognition have been recognized in the field and are, as such, not controversial. Examples are the modern understanding of motion as sensory-motor integration [36], the emphasis on complex, ecologically valid scenarios in behavioral studies [6, 24] or the focus on active sensing [1]. While these approaches emphasize interactions between the organism and its environment, another line of research analyses interactions within the organism. The brain is, of course, an organ, and as such it is embedded in biochemical, immunological, hormonal and mechanical processes in the body. Again, the interaction is bi-directional: the brain orchestrates physiological processes (e.g. through the autonomous nervous system), but it is also affected by these processes. A prominent recent example for such body-to-brain signaling is the increasing awareness for interactions between the gut microbiome and the brain, which may be important for normal cognition and behavior including impaired cognitive function in neuropsychiatric conditions and aging [26, 28].

Recently, a series of publications has shown that physiological processes in the body have immediate effects on the brain’s dynamic activity patterns. Many functions in organisms are cyclic, including most vegetative processes [29]. Similarly, most brain activity is organized in the form of coherent network oscillations at many different time scales [7]. Given the intricate connectivity between the brain and body, it is not far-fetched to search for mutual interactions of somatic and neuronal oscillations. Testing this global idea does, however, require detailed measurements and analyses. The most pressing questions are:

## Directionality

Do﻿ rhythms generated in neuronal networks govern somatic oscillations or vice versa? Obviously, central pattern generators like the brainstem respiratory networks suggest a brain-to-body causality. Recent findings, however, indicate several examples of body-to-brain signaling which affects neuronal rhythms. Impact of somatic rhythms on brain activity has been shown for respiration (reviewed in [16, 33]), heartbeat [2] and intestinal activity cycles [3]. It is now time to describe these interactions in detail and untangle their complex, probably bi-directional causality [20].

## Frequency

Somatic activity follows a wide range of frequencies, ranging from cycle times of ~ 1 s (heartbeat) to circadian, monthly and even circannual rhythms [29]. Neuronal network oscillations, on the other hand, cover mostly higher frequencies, beginning with ultrafast cortical oscillations at cycle lengths of 1–2 ms up to slow rhythms at tens of seconds [7, 8]. The most intensely studied cortical network oscillations range from delta (~ 1 Hz, 1 s) to fast gamma or ripples (~ 200 Hz, 5 ms, [13]). Thus, somatic and neuronal rhythms overlap, but do also cover distinct frequency ranges. It may be that slower brain rhythms are present but escape detection [4, 11]. Systematic and causal interactions between rhythms at different frequencies are, however, well feasible [21, 32]. A recent experimental example is the entrainment of theta-gamma coupling by respiration rate [15],see also [5, 40]. Given the notorious difficulty of slow or even DC recordings in EEG, researchers have looked for peripheral substitute signals to monitor somatic processes mediated via neuronal networks. In this Special Issue, Schaefer et al. [31] summarize published evidence for or against respiration-associated changes in pupil diameter. Their meta-analysis does, however, raise skepticism that this parameter provides any reliable readout signal for respiration-brain-coupling.

## Mechanisms

Causal interactions between two oscillators need a physical substrate. While brain-to-body signaling may mostly depend on established neuronal connections via autonomic (e.g. for heartbeat regulation) or somatic (e.g. for respiration) efferents, body-to-brain signaling seems to be more variable and less well analyzed. Sensory neuronal fibers clearly contribute to the entrainment of oscillations in the CNS, as, e.g., described for cardiac baroreceptors in this Special Issue [23]. Many studies, including those presented here, show how feedback from respiration shapes rhythmic brain activity, but the precise pathways are not entirely resolved. Nasal airflow seems to play an important role [37, 38], but it is not entirely clear whether thermal, mechanical or olfactory cues are decisive ([19], this Special Issue). Moreover, brainstem central patterns generators do also contribute to the synchronization of neuronal spiking activity to respiration [20]. Finally, immediate, non-neuronal signals may couple somatic processes to brain activity, including respiration-dependent changes in blood oxygenation ([39], this Special Issue) or breathing- or heartbeat-synchronous intracranial pressure changes [35].

## Function

Finally, and probably most importantly, we might ask what is the function, or biological relevance, of body-to-brain signaling. It seems obvious that central pattern generators for breathing or networks regulating autonomous function can make use of physiological feedback signals to adapt their activity to somatic functions, e.g. by terminating inspiration following input from pulmonary stretch receptors [30]. However, rhythmic activity in neuronal networks may constitute a functionally relevant signal by itself, supporting the generation of neuronal assemblies and the formation of complex multi-neuronal activity patterns [7, 13]. It is feasible that somatic feedback is simply ‘used’ as a synchronizing signal for brain oscillations in the same frequency domain [33]. Indeed, somatic feedback signals modulate brain activity across multiple different species, including humans ([22, 38], [14], this Special Issue), indicating that such effects are ecologically advantageous. They may facilitate cognitive operations like, e.g., perception, which does indeed depend on the respiratory cycle [22, 38]. In this Special Issue, Folschweiller and Sauer [12] describe how respiration entrains frontal cortical networks such that the formation and activation of neuronal assemblies is supported. Heck and Varga [17] provide an even broader framework for distributed brain activity which suggests that external (sensory) and internal (proprio- or interoceptive) signals contribute to the distributed, yet context-dependent activity patterns of the brain enabling adapted behavior. This view may posit somatic feedback in a much wider, integrative concept of physiology within the tradition of embodied cognition [3].

The Special Issue unites four comprehensive reviews on somatic feedback signals and brain function, covering its potential cognitive-behavioral significance [12, 17], the role of slow (respiratory) nasal feedback signals for brain dynamics [19] and the role of rhythmic oxygen pressure oscillations for potential neuronal effects of respiration [39]. Five original articles address specific effects of respiration on gamma oscillations [14], the state-dependence of respiration-cortical activity coupling [18], entrainment of brainstem neurons of the reticular formation by cardiac activity Kocsis and Topchyi [23], the molecular mechanisms linking hypoxia-mediated activation of carotid bodies to increased ventilation [25] and a new neuromodulatory effect on respiratory rhythm generation in brainstem networks by the peptide bombesin [27]. Finally, a systematic literature review summarizes the evidence for, or against, consistent changes of pupil diameter with respiration.

Together, this collection of articles by internationally renowned experts increases our knowledge and awareness for the close, bi-directional interactions between the brain and body. The Special Issue reminds us of the biological and ecological embedding of the brain which is by no means a physically detached information-processing machine.
